# Integrated single-cell and bulk RNA sequencing in pancreatic cancer identifies disulfidptosis-associated molecular subtypes and prognostic signature

**DOI:** 10.1038/s41598-023-43036-7

**Published:** 2023-10-16

**Authors:** Yunhao Wu, Jin Shang, Qiang Ruan, Xiaodong Tan

**Affiliations:** 1 Department of General Surgery, Shengjing Hospital of China Medical University, Shenyang, 110004 Liaoning China; 2 Department of Radiology, Shengjing Hospital of China Medical University, Shenyang, 110004 China; 3 Virology Lab, Shengjing Hospital of China Medical University, Shenyang, 110004 China

**Keywords:** Computational biology and bioinformatics, Cancer, Cancer microenvironment, Cancer models, Gastrointestinal cancer, Tumour biomarkers, Tumour immunology, Prognostic markers

## Abstract

Pancreatic cancer (PC) is known for its high degree of heterogeneity and exceptionally adverse outcome. While disulfidptosis is the most recently identified form of cell death, the predictive and therapeutic value of disulfidptosis-related genes (DRGs) for PC remains unknown. RNA sequencing data with the follow-up information, were retrieved from the TCGA and ICGC databases. Consensus clustering analysis was conducted on patient data using R software. Subsequently, the LASSO regression analysis was conducted to create a prognostic signature for foreseeing the outcome of PC. Differences in relevant pathways, mutational landscape, and tumor immune microenvironment were compared between PC samples with different risk levels. Finally, we experimentally confirmed the impact of DSG3 on the invasion and migration abilities of PC cells. All twenty DRGs were found to be hyperexpressed in PC tissues, and fourteen of them significantly associated with PC survival. Using consensus clustering analysis based on these DRGs, four DRclusters were identified. Additionally, altogether 223 differential genes were evaluated between clusters, indicating potential biological differences between them. Four gene clusters (geneClusters) were recognized according to these genes, and a 10-gene prognostic signature was created. High-risk patients were found to be primarily enriched in signaling pathways related to the cell cycle and p53. Furthermore, the rate of mutations was markedly higher in high-risk patients, besides important variations were present in terms of immune microenvironment and chemotherapy sensitivity among patients with different risk levels. DSG3 could appreciably enhance the invasion and migration of PC cells. This work, based on disulfidoptosis-related genes (DRGs), holds the promise of classifying PC patients and predicting their prognosis, mutational landscape, immune microenvironment, and drug therapy. These insights could boost an improvement in a better comprehension of the role of DRGs in PC as well as provide new opportunities for prognostic prediction and more effective treatment strategies.

## Introduction

Pancreatic cancer (PC) serves the most deadliest digestive neoplasm, with ductal adenocarcinoma being the predominating form and a five-year survival rate of approximately 12%^1^. Expected to rank as the 2nd lethal tumor by 2040 due to its high metastasis rates and limited treatment options, PC poses a significant challenge^2,3^.

Disulfidptosis is a novel form of cell death resulting from cytoskeletal collapse triggered by abnormal accumulation of intracellular disulfides^4^. The phenomenon has been named disulfidptosis by Gan and colleagues. Cells with high solute carrier family 7 member 11 (SLC7A11) expression transport large amounts of cystine, which is normally reduced to cysteine by glucose-generated nicotinamide adenine dinucleotide phosphate (NADPH), thus avoiding exposure to disulfide stress^5,6^. However, glucose starvation and NADPH deprivation lead to a build-up of intracellular disulfide bonds, including cystine and glutamyl-cystine, and the formation of abnormal disulfide bonds in actin cytoskeleton proteins, contributing to the breakdown of the actin network that eventually leads to cell death^4,5^. The relationship between disulfidptosis and diseases, especially cancer, is not yet fully understood, but subsequent research is expected to elucidate this theme.

Cells can be distinguished into regulated cell death (RCD) and accidental cell death based on their morphology, biochemistry, and function^7^. While apoptosis is the most well-known form of RCD, other types of RCD have been identified in recent years, including necroptosis, pyroptosis, ferroptosis, and cuproptosis^8,9^. RCD can impact the tumor microenvironment (TME) by liberating pathogenic or damage-associated molecular patterns (PAMPs or DAMPs), affecting cancer prognosis, progression, metastasis, immune surveillance, and the effectiveness of anticancer therapy^9–13^. Disulfidptosis is a newly discovered form of RCD, and it is currently unknown how it functions in PC development and treatment response. Therefore, this study aimed to preliminarily investigate how disulfidation affects prognosis, TME and treatment response in patients with PC.

In this study, we utilized DRGs to classify PC patients into four disulfidptosis subtypes and, on the basis of the differentially expressed genes (DEGs) of the disulfidptosis subtypes, discern ed four gene subtypes. A prognostic signature was then built to forecast the survival of patients and to delineate the entire landscape of immune infiltration in PC patients. This model is capable of stratifying PC patients based on their risk and is promising for predicting prognosis and guiding personalized treatment. Besides, we found that DSG3 could be a potential marker for PC, revealing a novel promising target for targeted therapy of PC.

## Methods

### Data source

The transcript sequencing data, mutation data, and clinical profiles for PC patients were obtained from the Cancer Genome Atlas (TCGA) (https://portal.gdc.cancer.gov/) and the International Cancer Genome Consortium (ICGC, PACA-AU cohort, available at https://dcc.icgc.org/). The gene expression matrices from these sources were merged to create a combined TCGA-ICGC dataset comprising 269 PC samples. The list of DRGs used in the analysis was obtained from the latest literature report^4^. To analyze the differential expression of DRGs between PC tissues and normal tissues, the Genotype-Tissue Expression (GTEx) data, sourced from the UCSC Xena website (https://xenabrowser.net/), was utilized. Additionally, the gene copy number profiles of PC samples were obtained from the same UCSC Xena database. In cases where multiple probes targeted a single gene, the average expression value was used for analysis. Furthermore, a single-cell RNA sequencing dataset, specifically the GSE155698 dataset, containing 16 PC samples, was downloaded from the GEO database.

### Differential expression analysis of DRGs

The expression of DRGs in PC was analyzed using the “limma” package in R. The Wilcoxon test method was employed to assess the significance of differential expression between groups. To evaluate the impact of DRG expression on overall survival (OS) in PC patients, Kaplan–Meier survival curves were generated. Additionally, network interaction analysis was conducted to better understand the relationships between DRGs using the “igraph” package in R.

### Consensus clustering analysis

We conducted consensus clustering analysis using the “Consensus Cluster Plus” R package to investigate the relationship between the expression of these DRGs and PC subtypes (20427518). The clustering variable (k) for the entire PC sample was selected to maximize the within-group similarity and minimize the between-group differences. Subsequently, we analyzed the survival differences between DR clusters and examined the expression differences of DRGs. Using the “limma” package, we identified DEGs between DR clusters, considering a log2 fold change (log2FC) greater than 1 and an adjusted *p*-value less than 0.05. Based on these DEGs, we performed consensus clustering analysis again to identify geneClusters. We visualized the expression of DEGs among geneClusters using a heatmap.

### Construction and validation of prognostic signature

Initially, the prognostic value of the DEGs was assessed using univariate Cox analysis, considering a significance threshold of *p*-value < 0.01. Subsequently, a prognostic signature was constructed using the eligible genes. The Least Absolute Shrinkage and Selection Operator (LASSO) regression, a high-dimensional predictive regression method, was employed to develop the PC prognostic signature. The “glmnet” package was utilized for this purpose. The risk score formula for the prognostic signature can be represented as:

$$\mathrm{Risk \,\, score}={\sum }_{\mathrm{i}}^{\mathrm{n}}\mathrm{Coffi}*\mathrm{ Expri}$$

We identified 13 PDAC samples in the combined TCGA-ICGC dataset for which survival data were not available. The combined TCGA-ICGC cohort, consisting of 256 PC samples, was randomly divided into two cohorts, namely the train and test cohorts, using the “createDataPartition” function from the “caret” package. The cohorts were created with 1000 randomizations to ensure robustness. Patients were categorized into two risk levels based on the median risk value as a baseline. The correspondence between DRclusters, geneClusters, risks, and survival states was visualized using the “ggalluvial” package to create a ggalluvial graph. Furthermore, we compared the risk scores of PC subtypes across DRclusters and geneClusters. To assess the predictive performance of our model, we utilized the “timeROC” package to plot ROC curves. Additionally, we employed 3D PCA to visualize the dispersion of samples from different risk levels.

### Cell type annotation

The scRNA-seq data were processed and analyzed using the “Seurat” package (v4.1.1) in R software. The data were created as seurat files for subsequent analysis. Sample integration was performed using the “Harmony” package (v0.1.0) to account for batch effects. To ensure data quality, low-quality cells were excluded based on specific criteria: (1) a minimum of 300 and a maximum of 7,000 RNA features (nFeature_RNA); (2) a minimum of 100,000 RNA counts (nCount_RNA); (3) a percentage of mitochondrial genes (percentage.mt) below 10%; and (4) a percentage of hemoglobin genes (percent.HB) below 1. After applying these screening criteria, a total of 34,758 cells were obtained. Data normalization was performed using the “NormalizeData” function, and 2000 highly variable genes were identified using the “FindVariableFeatures” function for subsequent PCA downsampling clustering analysis with a resolution of 1.0. Single-cell subpopulations were annotated using common cell marker genes.

### Analysis of the clinical significance of the prognostic signature

A nomogram was generated using the “rms” R package. Furthermore, we investigated the correlation between the risk score and clinical features. Additionally, we utilized the “oncoPredict” package to analyze the sensitivity of different risk patients to clinically available chemical drugs for PC. In order to gather comprehensive information about these drugs, we explored the 2D or 3D structures of these chemicals. The 2D/3D structures of the chemicals were obtained from the PubChem website (https://pubchem.ncbi.nlm.nih.gov/).

### Function enrichment analysis

To compare enriched signaling pathways across PC subtypes, we conducted the GSVA using the GSVA package. Additionally, we performed Gene Ontology (GO) and Kyoto Encyclopedia of Genes and Genomes (KEGG) enrichment analyses using the “clusterProfiler” package^14^. Lastly, we utilized the GSVA analysis to compare the differences in the signaling pathways enriched by patients in different risk categories.

### Mutation analysis

The frequency of mutations in DRGs among PC patients in the TCGA database was visualized using the “maftools” package. To compare the OS of PC patients with different TMB, we calculated TMB and analyzed its correlation with DRclusters and geneClusters using Spearman correlation analysis. Furthermore, the mutation patterns of patients with different risk levels were depicted using the “maftools” package.

### Analysis of tumor immune microenvironment

To investigate the variation in immune cell infiltration levels between PC subtypes, we employed the ssGSEA. Furthermore, we calculated the ESTIMATE score in PC tissue using the “estimate” package. The “CIBERSORT” algorithm was utilized to estimate the level of infiltrated immune cells in PC tissues, and we compared the level of infiltration between different risk groups of PC patients. In this study, we utilized the “GSVA” package to assess the active level of immune function in PC samples. Additionally, we examined the expression differences of immune checkpoints and HLA family molecules among patients with different risk levels.

#### Analysis of signature genes

The expression level of DSG3 in Pan-cancer was measured using the Wilcoxon rank sum test. We analyzed the association between DSG3 expression and clinical features of PC patients. Furthermore, we assessed the relationship between DSG3 expression and patient survival using the log-rank test. Additionally, we explored the genes that are associated with DSG3. Finally, we analyzed the relationship between DSG3 and the extent of immune cell infiltration using the ssGSEA.

#### Tissue specimens and cell lines

Four cases of fresh PC tissues and their paired paraneoplastic tissues were obtained from Shengjing Hospital of China Medical University to ascertain DSG3 expression level in PC tissues. PC samples were well preserved in liquid nitrogen. The National Collection of Authenticated Cell cultures (Shanghai, China) provided the BxPC-3 and PANC-1 cells, which were maintained in DMEM complete medium. This research was approved by the Ethics Committee of Shengjing Hospital of China Medical University (2022PS168K). All experiments were conducted in strict accordance with the relevant guidelines and regulations of the Ethics Committee of Shengjing Hospital of China Medical Universityand performed in accordance with the Declaration of Helsinki. The informed consent was obtained from all participants and/or their legal guardians.

#### Western blot

As stated in the earlier protocol, the western blot (WB) was manipulated^15^. The primary antibodies utilized were anti-DSG3 (1:500, ZENBIO, Chengdu, China) and anti-β-actin (1:10,000, Proteintech, USA). The HRP-conjugated Affinipure Goat Anti-Rabbit IgG(H + L) (1:5,000, Proteintech, USA) was utilized as secondary antibody.

#### Cell transfection

DSG3 short hairpin RNA (shDSG3) and DSG3 overexpression plasmid were designed by GeneChem (Shanghai, China). The target sequence of shDSG3 is 5′-CACCGGACGTAACGATGGTGGATACCGAAGTATCCACCATCGTTACGTCC-3′. The component sequence of DSG3 overexpression plasmid is CMV enhancer-MCS-polyA-EF1A-zsGreen-sv40-puromycin. Cells were transfected following the manufacturer's protocols of Lipofectamine 3000 (Invitrogen, Carlsbad, CA).

#### Invasion and migration assays

The wound healing and the transwell experiments were performed to evaluate cellular migration and invasion capacity. Cells received different treatments were digested and inoculated in 6-well plates for wound healing assays. After the cell density reached approximately 95%, a straight line was drawn over the surface of each well with a 100 μL sterile pipette. At 0 and 24 h, respectively, the wound area was photographed with the inverted microscope (Nikon DS-RI2, Japan). We then performed a permeabilization assay by adding 800 μL of medium containing 10% fetal bovine serum (Corning Incorporated, USA) to the lower chamber and 200 μL of serum-free medium with 20,000 cells to the upper chamber. After 24 h of incubation, cells that had crossed the membrane were fixed with 4% paraformaldehyde and stained with crystal violet. Cell images were taken with an inverted microscope at 200 × magnification.

#### Statistical analysis

This project was conducted with R software (https://www.r-project.org/, version 4.2.1) and their corresponding packages. The TCGA-ICGA integrated dataset was randomly grouped by the “createDataPartition” function of the “caret” package. The Wilcoxon test was employed for gene differential expression analysis, and Kaplan–Meier survival analysis was estimated by log-rank test. The correlation analysis was performed by the “spearman” function. *P* value < 0.05 was deemed as statistically significant unless specifically mentioned. ***: < 0.001; **: < 0.01; *: < 0.05; ns: > 0.05.

### Ethics approval and consent to participate

This research was approved by the Ethics Committee of Shengjing Hospital of China Medical University (2022PS168K). All experiments were conducted in strict accordance with the relevant guidelines and regulations of the Ethics Committee of Shengjing Hospital of China Medical Universityand performed in accordance with the Declaration of Helsinki. The informed consent was obtained from all participants and/or their legal guardians.

## Results

### Genetic and transcriptional alterations of DRGs

**Figure 1:**
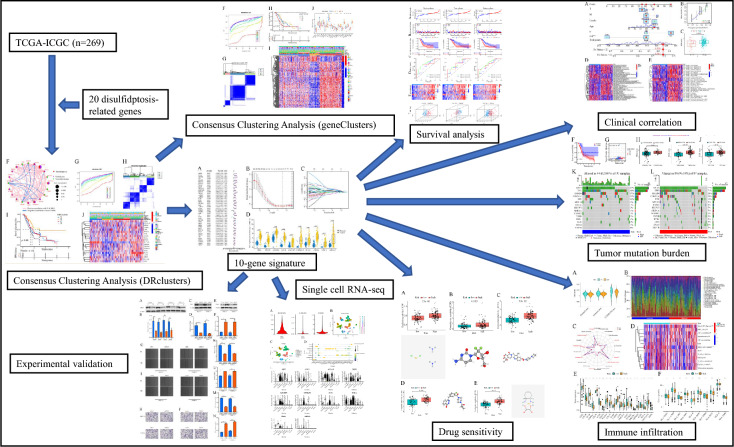
The workflow of this study.

**Figure 2:**
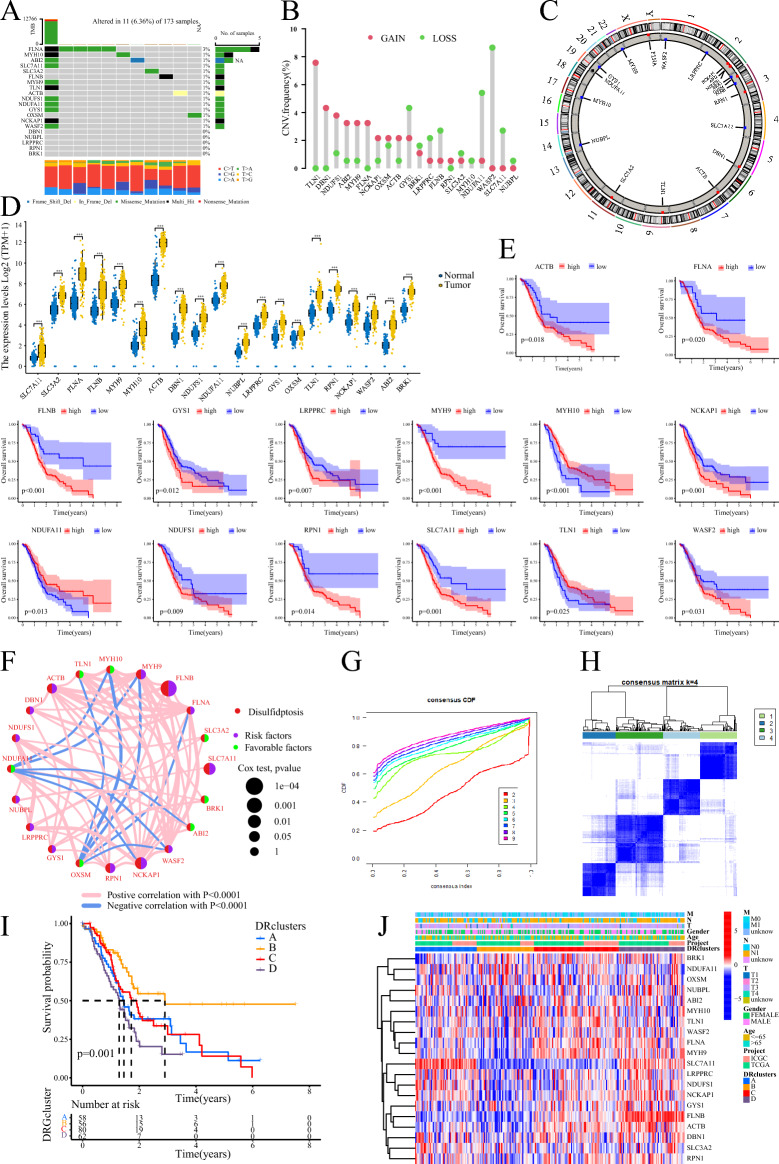
Genetic and transcriptional alterations of DRGs and clustering analyses of DRGs. (**A**) Waterfall plot of DRGs in TCGA. (**B**, **C**) The frequency of occurrence of CNV in DRGs and the corresponding position on the chromosome. (**D**) Expression levels of DRGs in PC tissues and normal tissues. (**E**) Kaplan–Meier survival curve analysis of DRGs in PC. (**F**) Interrelationships between DRGs. (**G**, **H**) Consensus clustering analysis based on DRGs to obtain DRclusters indicated that the optimal number of subtypes was 4. (**I**) Kaplan–Meier survival curve analysis of DRclusters. (**J**) Heatmap of DRGs expression in DRclusters.

Our study workflow is depicted in Fig. 1, while Table 1 presents general information about the twenty DRGs. The PC dataset, comprising 179 PC samples and four normal pancreatic tissue samples, was collected from the TCGA database. Additionally, the ICGC-AU cohort provided 91 PC samples. Analysis using a waterfall plot revealed a generally low mutation frequency of the DRGs in PC tissues, with the FLNA gene exhibiting the highest mutation frequency of only 3% (Fig. 2A). However, the analysis of somatic copy number alterations (CNV) indicated that all 20 DRGs exhibited CNV, with the TLN1, WASF2, and NDUFA11 genes showing extensive CNV (Fig. 2B). Figure 2C displays the location of the DRGs on their corresponding chromosomes where CNV occurs. Moreover, all 20 DRGs were found to be significantly upregulated in PC tissues (Fig. 2D), and most of them were associated with the prognosis of PC patients. High expression levels of ACTB, FLNA, FLNB, GYS1, LRPPRC, MYH9, NCKAP1, NDUFS1, RPN1, SLC7A11, and WASF2 were notably related to a poor outcome for PC patients. Conversely, high expression levels of MYH10, NDUFA11, and TLN1 implied better survival rates (Fig. 2E). Furthermore, a correlation network diagram revealed strong interconnections among these DRGs, indicating potential regulatory relationships between them (Fig. 2F).

**Table 1:** The general information about the DRGs.

Gene	Full name
SLC7A11	Solute carrier family 7 member 11
SLC3A2	Solute carrier family 3 member 2
FLNA	Filamin A
FLNB	Filamin B
MYH9	Myosin heavy chain 9
MYH10	Myosin heavy chain 10
TLN1	Talin 1
ACTB	Actin beta
DBN1	Drebrin 1
NDUFS1	NADH:ubiquinone oxidoreductase core subunit S1
NDUFA11	NADH:ubiquinone oxidoreductase subunit A11
NUBPL	NUBP iron-sulfur cluster assembly factor, mitochondrial
LRPPRC	Leucine rich pentatricopeptide repeat containing
GYS1	Glycogen synthase 1
OXSM	3-oxoacyl-ACP synthase, mitochondrial
RPN1	Ribophorin I
NCKAP1	NCK associated protein 1
WASF2	WASP family member 2
ABI2	Abl interactor 2
BRK1	BRICK1 subunit of SCAR/WAVE actin nucleating complex

### Identification of disulfidptosis subtypes

**Figure 3:**
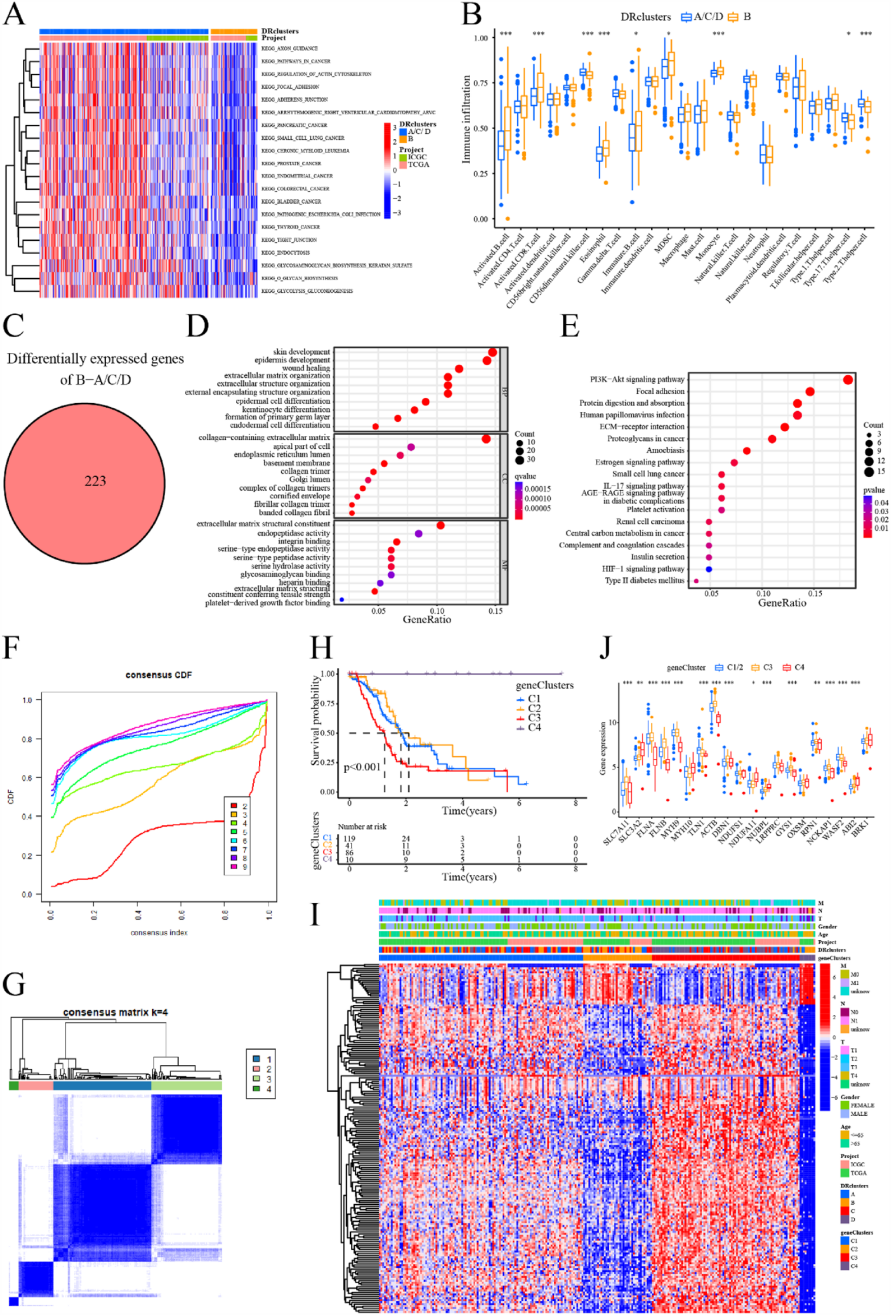
Obtain the DEGs between DRclusters and identify geneClusters. (**A**) GSVA analysis of DRclusters. (**B**) Analysis of immune cell infiltration in DRclusters by ssGSEA. (**C**) Identification of DEGs between DRclusters. (**D**, **E**) GO/KEGG enrichment analysis of DEGs. (**F**, **G**) Consensus clustering analysis based on DEGs to obtain geneClusters indicated that the optimal number of subtypes was 4. (**H**) Kaplan–Meier survival curve analysis of geneClusters. (**I**) Heatmap of DEGs expression in geneClusters. (**J**) Expression levels of DRGs in geneClusters. ***: < 0.001; **: < 0.01; *: < 0.05; ns: > 0.05.

To investigate the relationship between DRGs expression with PC subtype, we applied consensus clustering analysis to samples in the TCGA-ICGC integrated dataset. As the clustering variable (k) increased from 2 to 9, the strongest intra-subtype relatedness and the weakest inter-subtype relatedness were obtained where k = 4, implying that all PC sufferers should be optimally classified into four subtypes according to the 20 DRGs (Fig. 2G,H). Furthermore, patients in cluster B showed significantly improved OS compared to those in the other three subtypes (clusters A/C/D, Fig. 2I). Considering the similarity in survival time among patients in clusters A/C/D, we grouped them together as one subset of subtypes for comparison with cluster B. Additionally, the expression levels of the 20 DRGs were generally lower in cluster B compared to the other three clusters (Fig. 2J). We performed enrichment analysis using GSVA, which indicated that clusters A/C/D were enriched in signaling pathways related to cancer (including PC) and regulation of the actin cytoskeleton, among others (Fig. 3A). On the other hand, based on ssGSEA for immune cell infiltration, cluster B exhibited significantly higher levels of activated B cells, myeloid-derived suppressor cells (MDSCs), CD8 + T cells, and monocytes in PC tissue, whereas cluster A/C/D showed higher infiltration levels of CD56dim NK cells, Th17 cells, and Th2 cells (Fig. 3B). Moreover, we identified a total of 223 DEGs between cluster B and cluster A/C/D (Fig. 3C). These DEGs were predominantly enriched in processes related to wound healing, extracellular matrix organization, and signaling pathways such as PI3K-Akt and HIF-1 (Fig. 3D,E).

### Identification of gene subtypes

Following the identification of 223 DEGs between cluster B and cluster A/C/D (Table S1), we performed a consensus clustering analysis on PC patients once again. The strongest intra-subtype correlations were observed when k = 4 (Fig. 3F,G), resulting in the identification of four geneClusters. Notably, patients in cluster C3 exhibited a significantly worse prognosis compared to the other three subtypes (clusters C1/2/4), while patients in cluster C4 had the best prognosis (Fig. 3H). Considering the similarity in survival times between patients in clusters C1 and C2, these subtypes were also combined for comparison with cluster C3 or C4. We observed higher expression levels of the DEGs in cluster A/C/D of DRclusters and cluster C3 of geneClusters, which were associated with a worse prognosis (Fig. 3I). Furthermore, the expression of DRGs differed significantly among these three subtype cohorts. Specifically, SLC7A11, FLNA, FLNB, ACTB, DBN1, NUBPL, GYS1, RPN1, and NCKAP1 were significantly overexpressed in cluster C3, while only TLN1 was underexpressed. Most DRGs were expressed at the lowest level in the C4 cluster, while SLC3A2, MYH10, NDUFA11, NUBPL, OXSM, and ABI2 exhibited the highest expression levels in cluster C4 (Fig. 3J).

### Construction of prognostic signature

**Figure 4:**
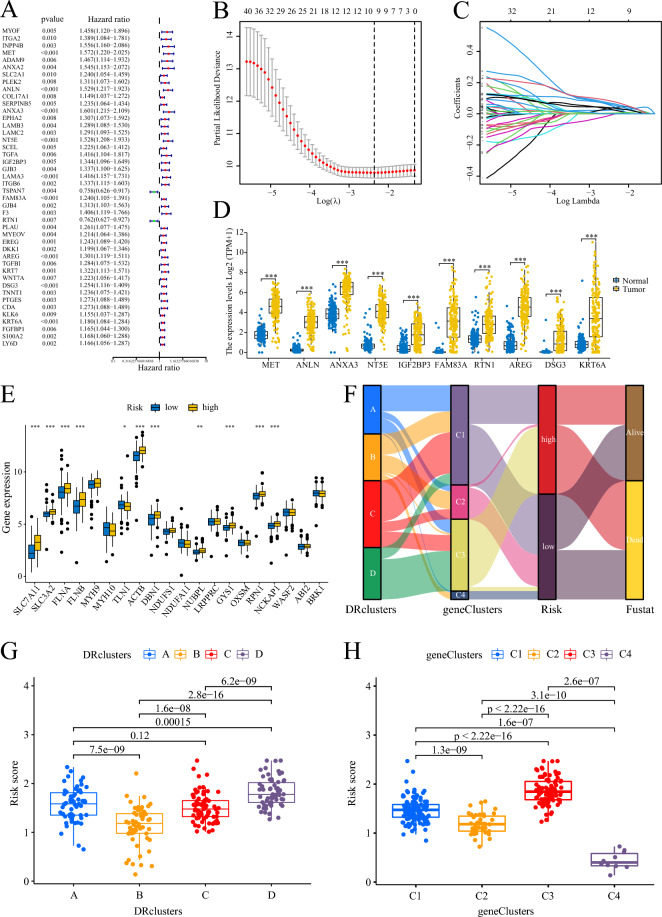
Construction of prognostic signature. (**A**) Identification of DEGs associated with PC prognosis by univariate Cox analysis. (**B**, **C**) LASSO regression analysis for screening 10 optimal genes. (**D**) Expression levels of the 10 signature genes in PC tissues and normal tissues. E Expression levels of DRGs in PC patients with different risks. (**F**) Ggalluvial showing the association between clusters, risk, and survival status. (**G**, **H**) Scatter plots showing the relationship between risk score with DRclusters and geneClusters. ***: < 0.001; **: < 0.01; *: < 0.05; ns: > 0.05.

Two hundred and fifty-six PC patients from the TCGA-ICGC integrated dataset were included in the study, and their corresponding survival information was used for model construction. Initially, univariate Cox regression analysis was performed to identify 44 prognostic-related DEGs for further analysis (Fig. 4A). Out of these 44 genes, all except TSPAN7 (HR 0.758) and RTN1 (HR 0.762) were identified as risk factors for PC survival. Conversely, TSPAN7 and RTN1 were found to be protective factors for PC. The TCGA-ICGC combined dataset was randomly divided into train and test cohorts, each comprising 128 PC samples. Subsequently, a 10-gene prognostic signature was derived based on the optimal λ value using the Lasso regression method (Fig. 4B,C). The prognostic signature model is obtained by calculating the sum of the products of the Lasso coefficient and the expression of the 10 genes, as follows:

$$ \begin{aligned} {\text{Risk}}\;{\text{score}} & = \left[ {0.0244{\text{* Expr}}.{\text{ MET}}} \right] + { }\left[ {0.0405{\text{* Expr}}.{\text{ ANLN}}} \right] + { }\left[ {0.0639{\text{ * Expr}}.{\text{ ANXA}}3} \right] \\ & \quad + { }\left[ {0.0238{\text{* Expr}}.{\text{ NT}}5{\text{E}}\left] {{ } + { }} \right[0.0445{\text{* Expr}}.{\text{ IGF}}2{\text{BP}}3\left] {{ } + { }} \right[0.0053{\text{ * Expr}}.{\text{ FAM}}83{\text{A}}} \right] \\ & \quad + { }\left[ { - 0.0015{\text{ * Expr}}.{\text{ RTN}}1} \right] + \left[ {0.0553{\text{* Expr}}.{\text{ AREG}}} \right] \\ & \quad { + }\left[ {0.0770{\text{* Expr}}.{\text{ DSG}}3} \right]{ + }\left[ {0.0360{\text{ * Expr}}.{\text{ KRT}}6{\text{A}}} \right] \\ \end{aligned} $$

**Figure 5:**
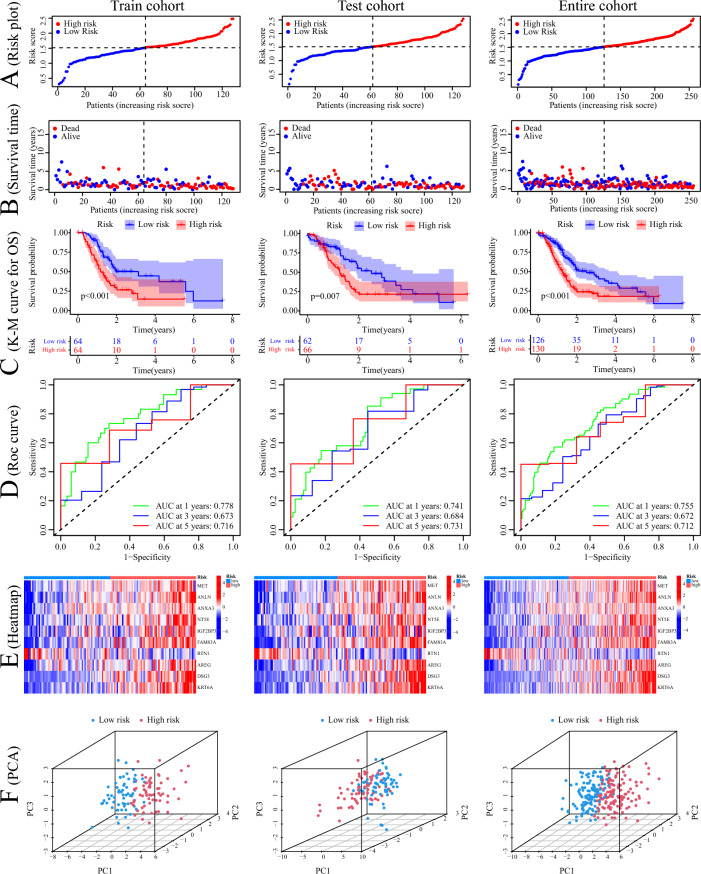
Construction and verification of prognostic signature. (**A**, **B**) Distribution of the risk score and survival time in the train cohort, test cohort, and entire cohort. (**C**) Kaplan–Meier survival curve analysis of risk score in the train cohort, test cohort, and entire cohort. (**D**) ROC analysis of this signature in predicting the 1-, 3-, and 5- year survival of patients with PC in the train cohort, test cohort, and entire cohort. (**E**) Heatmap of the expression of signature genes in patients with high- and low-risk in the train cohort, test cohort, and entire cohort. (**F**) PCA plots in 3D of the distribution of different risk samples in the train cohort, test cohort, and entire cohort.

All 10 signature genes exhibited high expression levels in PC tissues (Fig. 4D), and most of the DRGs, including SLC7A11, SLC3A2, FLNA, FLNB, TLN1, ACTB, DBN1, NUBPL, GYS1, RPN1, and NCKAP1, were upregulated in high-risk patients (Fig. 4E). The ggalluvial plot demonstrated minimal overlap between the high-risk cluster C3 and the low-risk cluster B, which exhibited the best prognosis (Fig. 4F). Cluster B predominantly consisted of low-risk samples, while cluster C3 mainly consisted of high-risk samples, and all patients in cluster C3 had deceased. DRcluster B had the lowest risk score, whereas geneCluster C3 had the highest risk score, aligning with their respective prognosis (Fig. 4G,H). PC patients were classified into two risk groups based on the median risk score, and distinct outcomes were observed between the groups (Fig. 5A,B). The low-risk group exhibited significantly better outcomes (Fig. 5C). To assess the efficiency of the model, the ROC curve was utilized, yielding area under the ROC curve (AUC) values of 0.778, 0.673, and 0.716 for predicting 1, 3, and 5 years of survival, respectively, in the train cohort. In the test cohort, the AUC values were 0.741, 0.684, and 0.731, while in the entire cohort, they were 0.755, 0.672, and 0.712 (Fig. 5D). The heatmap analysis revealed that only RTN1 was upregulated in the low-risk group, while the other 9 genes were more highly expressed in the high-risk group (Fig. 5E). Finally, PCA demonstrated that samples from different risk groups were independent of each other (Fig. 5F).

### Cell types expressing signature genes

**Figure 6:**
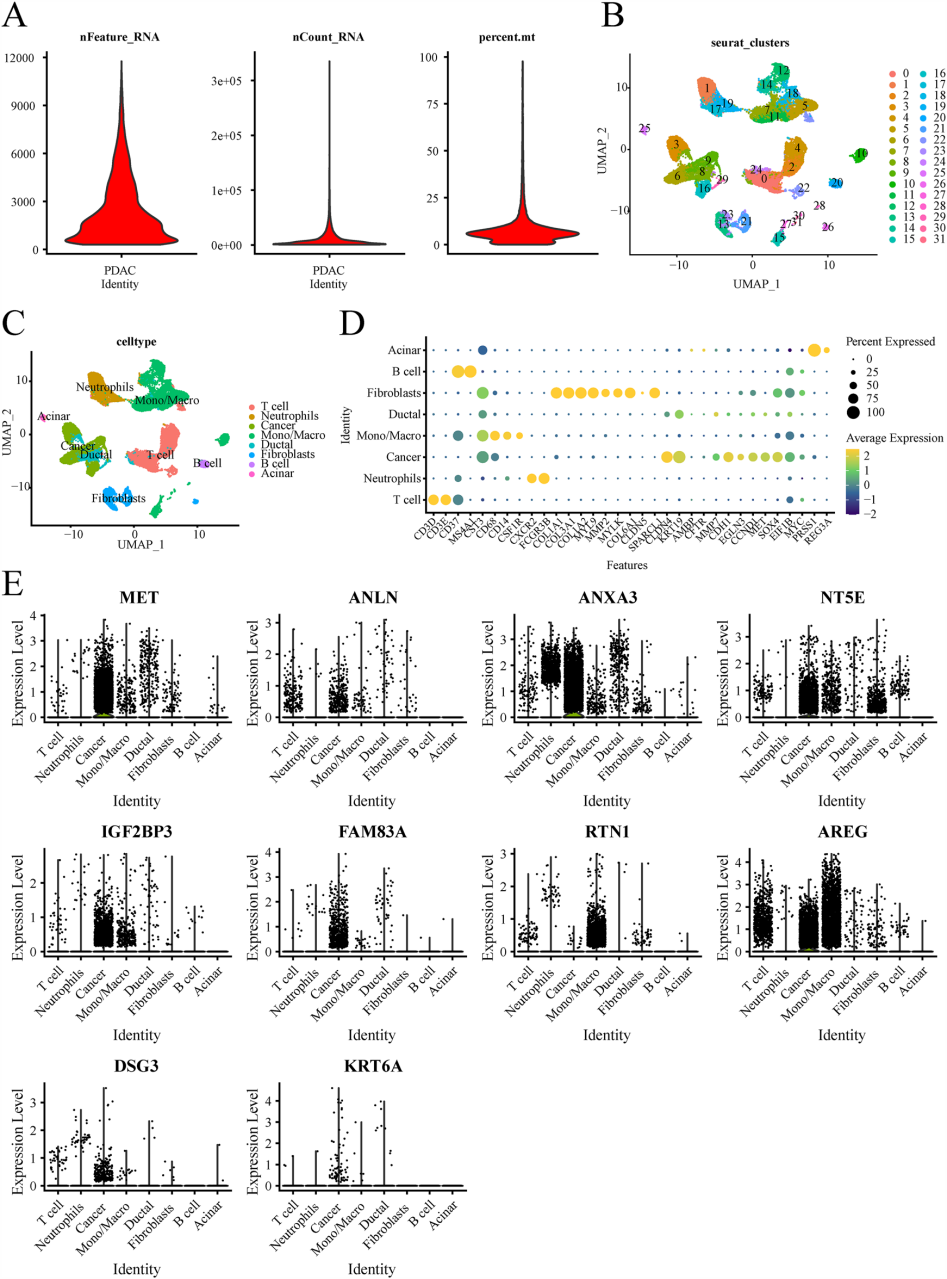
(**A**) Violin plots show the number of genes (nFeature), the sequence count per cell (nCount), and percentage of mitochondrial genes (per cent.mt). (**B**) The UMAP plot of cell clusters. (**C**) The UMAP plot of celltypes. (**D**) The bubble plot of marker gene for cell types. (**E**) The violin plot shows that the cell types expressing signature genes.

Cells were subjected to standard screening procedures, resulting in the identification of 24,722 genes in 34,758 cells. Violin plots were generated to visualize various characteristics, including the number of genes (nFeature), the sequence count per cell (nCount), and the percentage of mitochondrial genes (percent.mt) (Fig. 6A). Initially, all cells were clustered into 32 distinct clusters. Subsequently, the cells were annotated and assigned to 8 different cell types, namely acinar cells, B cells, fibroblasts, ductal mono/macro, cancer, neutrophils, and T cells (Fig. 6B–D). The violin plot analysis revealed that most of the signature genes were predominantly expressed in cancer cells, with the exception of RTN1 and AREG, which exhibited higher expression in monocytes/macrophages (Fig. 6E).

### Clinical value of signature

**Figure 7:**
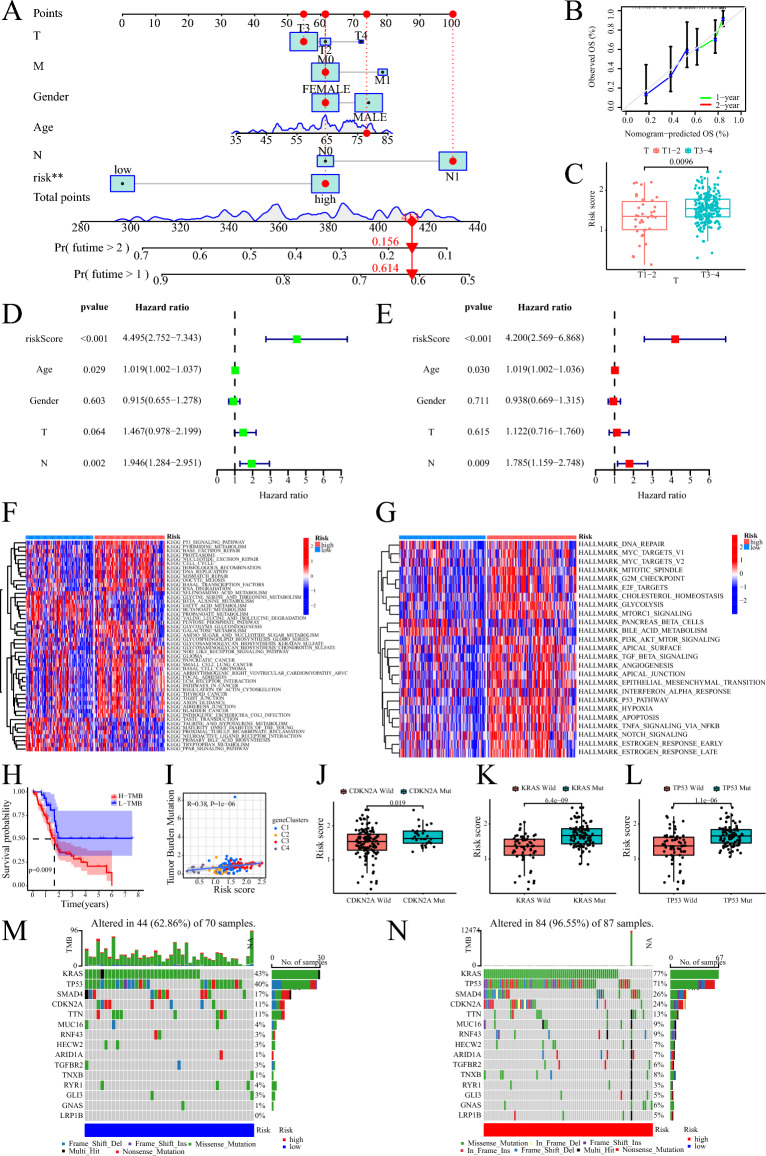
The clinical application value of risk score and gene mutation. (**A**) The nomogram of the risk score and clinical parameters (age, gender, and TNM stage) in TCGA cohort. (**B**) The calibration curves reflect the degree of accuracy of the nomogram. (**C**) Correlation between risk score and T-stage. (**D**) Prognostic value of univariate Cox analysis of risk score. (**E**) Prognostic value of multivariate Cox analysis of risk score. (**F**) GSVA demonstrating risk score of KEGG signaling pathway enrichment. G GSVA demonstrating risk score of Hallmark signaling pathway enrichment. (**H**) Kaplan–Meier survival curve analysis of high TMB and low TMB. (**I**) The relationship between TMB with risk score and geneClusters. (**J**–**L**) The relationship between risk score and mutations in PC driver genes (CDKN2A, KRAS, and TP53). (**M**) Waterfall map of gene mutations in patients of low risk group. (**N**) Waterfall map of gene mutations in patients of high risk group.

To assess the potential clinical utility of our risk score, we constructed a nomogram to predict the 1-year and 2-year OS of patients. The nomogram demonstrated favorable predictive performance, indicating its usefulness as a prognostic tool (Fig. 7A,B). Additionally, we observed that patients with T3-4 stage had higher risk scores compared to those with T1-2 stage, suggesting a correlation between the risk score and tumor stage (Fig. 7C). Furthermore, we found that the risk score could serve as an independent prognostic factor for PC patients, irrespective of other clinical characteristics (Fig. 7D,E).

### Functional enrichment of prognostic signature

The KEGG enrichment analysis using the GSVA revealed that high-risk patients exhibited prominent enrichment in several signaling pathways, including P53, cell cycle, DNA replication, PC, and regulation of the actin cytoskeleton. Conversely, the peroxisome proliferator-activated receptor (PPAR) pathway was significantly upregulated in low-risk patients (Fig. 7F). Similarly, the cancer hallmark enrichment analysis utilizing the GSVA demonstrated that high-risk patients exhibited enrichment in signaling pathways such as mammalian target of rapamycin complex 1 (mTORC1), PI3K-AKT-mTOR, transforming growth factor beta (TGFβ), epithelial-mesenchymal transition, P53, and Notch (Fig. 7G).

### Analysis of genetic variation

Upon calculating the TMB of PC patients, two mutation groups were identified. Patients in the high mutation group exhibited shorter survival compared to those in the low mutation group (Fig. 7H). Furthermore, TMB showed a positive association with patient risk, with geneCluster C3 demonstrating the highest TMB and risk score among the clusters (Fig. 7I). The risk score displayed a strong correlation with the frequency of variants in PC driver genes, including CDKN2A, KRAS, and TP53. Patients harboring mutations in CDKN2A, KRAS, and TP53 displayed notably higher risk scores compared to those with wild-type genes (Fig. 7J–L). The mutation waterfall plots revealed a higher frequency of mutations in genes among high-risk patients, particularly in KRAS (77% vs. 43%), TP53 (71% vs. 40%), and CDKN2A (24% vs. 11%) (Fig. 7M,N).

### Relationship of prognostic signature to TME

**Figure 8:**
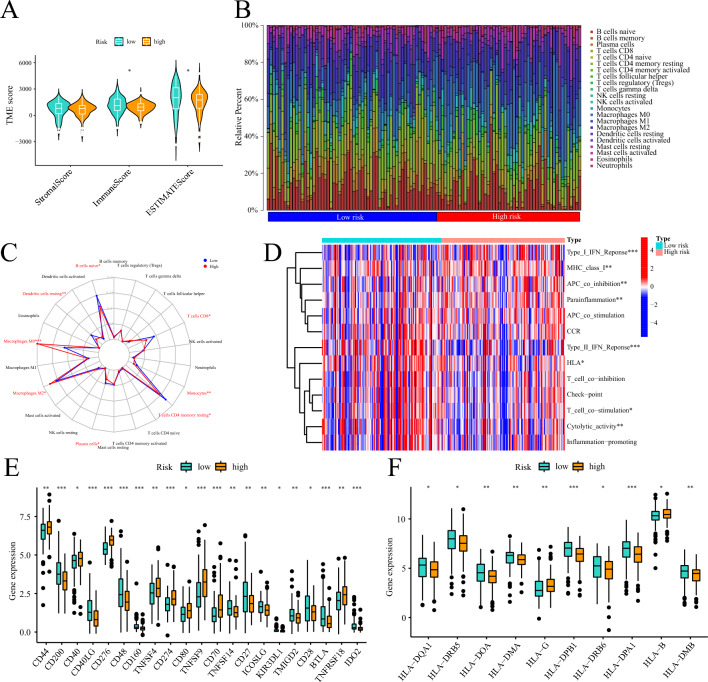
The relationship between risk score and PC immune infiltration pattern. (**A**) Violin diagram showing the ESTIMATE score of the PC samples. (**B**) Proportion of the infiltration of 22 immune cells in patients with different risks. (**C**) Radar plot showing the difference in the infiltration level of immune cells in patients with different risks. (**D**) Active differences in immune function in patients with different risks. (**E**) Differences in the expression levels of immune checkpoints in patients with different risks. (**F**) Differences in the expression levels of HLA family molecules in patients with different risks. ***: < 0.001; **: < 0.01; *: < 0.05; ns: > 0.05.

The high-risk group exhibited a significant reduction in both the immune score and composite ESTIMATE score compared to the low-risk group (Fig. 8A). There was a notable difference in the percentage of immune cell infiltration among samples with different risk levels (Fig. 8B). In low-risk patients, higher infiltration levels of B cells naïve, plasma cells, T cells CD4 memory resting, T cells CD8, dendritic cells resting, and monocytes were observed, whereas high-risk patients showed a higher proportion of macrophage M0 and M2 (Fig. 8C). The level of immune function activity varied significantly among samples from different risk groups. Immune functions such as MHC class I, parainflammation, APC co-inhibition, and Type I IFN response were less active in the low-risk group, while T cell co-stimulation, cytolytic activity, Type II IFN response, and HLA were more active (Fig. 8D). Dysregulation in the expression of several immune checkpoints was observed in different risk groups, including PD-L1 (also known as CD274), which was overexpressed in high-risk individuals (Fig. 8E). Additionally, the expression levels of certain HLA family molecules exhibited marked differences among patients with different risk levels (Fig. 8F).

#### Therapeutic value of prognostic signature

**Figure 9:**
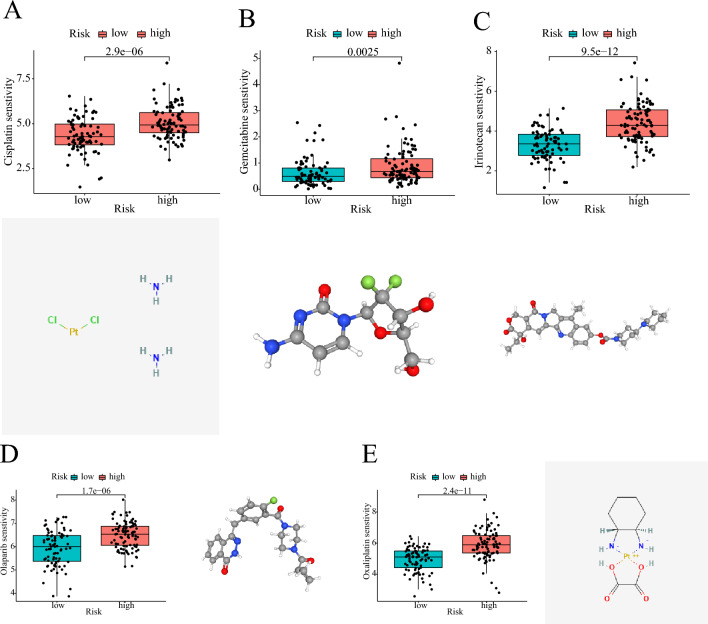
Relationship between risk score and chemotherapy drug sensitivity. (A) Low-risk patients are more sensitive to cisplatin. (**B**) Low-risk patients are more sensitive to gemcitabine. (**C**) Low-risk patients are more sensitive to irinotecan. D Low-risk patients are more sensitive to olaparib. E Low-risk patients are more sensitive to oxaliplatin.

Chemotherapy plays a vital role in the treatment of PC. In this study, we assessed the differences in risk scores and the sensitivity of PC patients to clinically available chemotherapy drugs. We identified five chemicals that displayed notable differences in sensitivities between high-risk and low-risk patients, and we provided their 2D or 3D chemical structures (Fig. 9). Low-risk patients exhibited significantly higher sensitivity to cisplatin, gemcitabine, irinotecan, olaparib, and oxaliplatin compared to high-risk patients. Consequently, these drugs may be more suitable for low-risk patients and have the potential to yield improved therapeutic outcomes.

#### The expression and function of DSG3 in PC

**Figure 10:**
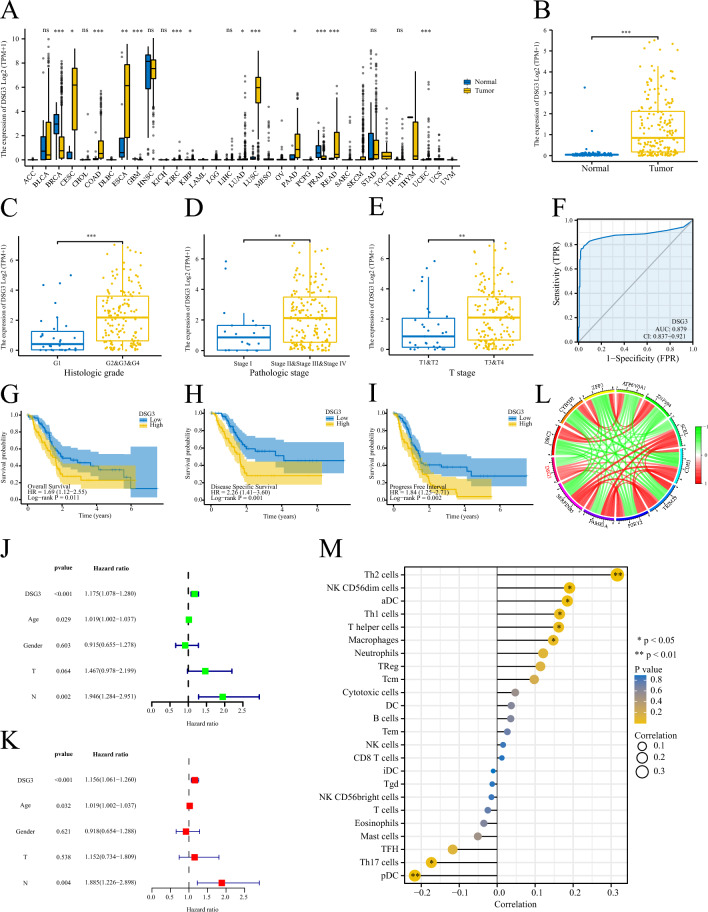
Analysis of the role of DSG3 in PC. (**A**) Expression of DSG3 in Pan-cancer. (**B**) Expression of DSG3 in PC and normal tissues. (**C**–**E**) Higher DSG3 expression level was correlated with higher histological grade, pathological stage, and T-stage of PC patients. (**F**) ROC curve of DSGs diagnosing PC patients. (**G**–**I**) High expression of DSG3 was associated with worse OS, DSS, and PFI in PC patients. OS: overall survival, DSS: disease-specific survival, and PFI: progression-free interval. (**L**) The chord plot shows the genes associated with DSGs. (**J**, **K**) Univariate and multivariate Cox regression analyses of DSG3 in PC. (**M**) ssGSEA analysis of the relationship between DSG3 with the level of immune cell infiltration in PC. ***: < 0.001; **: < 0.01; *: < 0.05; ns: > 0.05.

**Figure 11:**
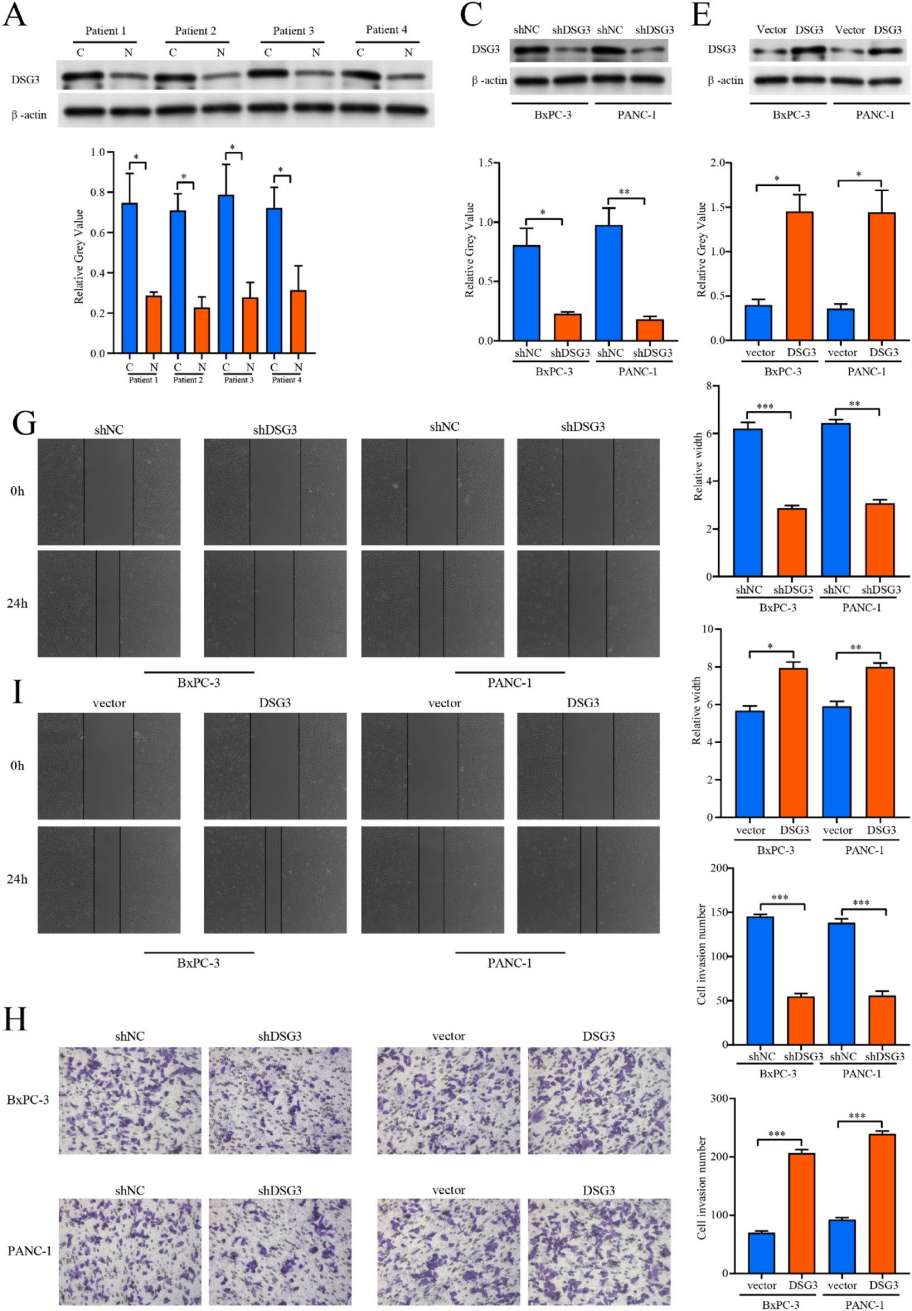
Experiments to verify the effect of DSG3 on PC migration and invasion. (**A**, **B**) The western blot showed that the expression level of DSG3 was higher in PC tissues than in normal tissues. C: Cancer tissue, N: Normal tissue. (**C**, **D**) The western blot verified the knockdown efficiency of shDSG3 in BxPC-3 and PANC-1 cells. (**E**, **F**) The western blot verified the overexpression efficiency of DSG3 overexpression plasmid in BxPC-3 and PANC-1 cells. (**G**, **K**) Wound healing assay verified that the migration ability of BxPC-3 and PANC-1 cells was reduced after DSG3 knockdown. (**I**, **L**) Wound healing assay verified that the migration ability of BxPC-3 and PANC-1 cells was increased after DSG3 overexpression. (**H**, **M**) Transwell assay verified that the invasive ability of BxPC-3 and PANC-1 cells reduced after DSG3 knockdown. (**J**, **N**) Transwell assay verified that the invasive ability of BxPC-3 and PANC-1 cells increased after DSG3 overexpression. ***: < 0.001; **: < 0.01; *: < 0.05; ns: > 0.05.

We conducted a comprehensive review of the available research on signature genes and investigated the correlation between these genes and the clinical traits of PC patients. Among these genes, DSG3, which has not been extensively studied in PC, exhibited a strong association with the clinical stage and prognosis of PC patients. We observed significant differential expression of DSG3 in pan-cancer, particularly in PC tissues (Fig. 10A,B), and higher expression levels of DSG3 were correlated with higher histological grade, pathological stage, and T-stage (Fig. 10C–E). The AUC value for the diagnosis of PC patients using DSG3 was 0.879 (CI 0.837–0.921, Fig. 10F). Furthermore, high expression of DSG3 was associated with poor disease-specific survival (DSS), OS, and progression-free interval (PFI) in PC patients (Fig. 10G–I). DSG3 was identified as an independent risk indicator for PC patients through both univariate and multivariate Cox regression analyses (Fig. 10J,K). Additionally, DSG3 showed a strong positive correlation with the expression of SCEL, CDH3, TRIM29, P2RY2, FAM83A, and SERPINB5, while exhibiting a negative association with ZNF594, ATP6V0A1, ZFP3, CYB5D2, and DRC3 (Fig. 10L). DSG3 appeared to be negatively associated with plasmacytoid dendritic cells (pDCs) and T-helper 17 (Th17) cells, while positively correlated with T cells, activated dendritic cells (aDCs), and macrophage infiltration (Fig. 10M). Given the significant role of DSG3 in PC, we conducted additional studies to examine its impact on the invasive and migratory capabilities of PC cells. We found that DSG3 expression was significantly elevated in PC tissues compared to adjacent noncancerous tissues (Fig. 11A,B). Silencing DSG3 using DSG3-shRNA in BxPC-3 and PANC-1 cells led to a noticeable reduction in DSG3 protein expression levels (Fig. 11C,D). Conversely, transfection of PC cells with a DSG3 overexpression plasmid resulted in increased DSG3 protein expression levels (Fig. 11E,F). Wound healing and transwell assays demonstrated a significant inhibition of invasion and migration in PC cells following DSG3 silencing (Fig. 11G,H,K,M). Conversely, excessive expression of DSG3 dramatically enhanced the invasion and migration capacities of PC cells (Fig. 11I,J,L,N).

## Discussion

PC is a major global health concern, ranking seventh (4.7%) in the world for cancer-related causes of death. Unfortunately, the incidence of PC is on the rise and it is associated with terrible outcomes^16^. Recent work by Gan et al. has suggested a new kind of cell death called disulfidptosis. This process involves the collapse of the actin network due to the excessive accumulation of disulfide bond molecules in the cell, ultimately leading to cell death^4^. Although the key gene SLC7A11, which is involved in disulfidptosis, shows significant high expression in most tumors, the relationship between disulfidptosis and cancer is insufficiently understood. Hence, this study aims to explore the potential associations between DRGs and molecular subtypes, prognosis, gene mutations, TME infiltration, and chemotherapy sensitivity in PC.

In this study, we identified four distinct DRclusters based on 20 DRGs. Patients in subtypes A/C/D had similar prognosis and DRGs expression patterns, while those in subtype B had longer survival times, lower DRG expression levels, and lower activity of cancer-related signaling pathways. Immune cell infiltration levels were also distinct in the two disulfidptosis populations, with activated B cells and activated CD8 T cells showing higher levels of infiltration in subtype B. DEGs between the two disulfidptosis populations were associated with the PI3K-Akt and HIF-1 signaling pathways. We subsequently identified four geneClusters based on DEGs, which were highly expressed in prognostically poor subtypes of DRclusters and geneClusters, suggesting that these DEGs may predict PC prognosis and immune infiltration. A 10-gene PC prognostic signature was developed based on DEGs using univariate Cox and LASSO regression. More than half of the 20 DRGs were highly expressed in high-risk PC patients, and patients with higher T-stage had higher risk scores, suggesting that some DRGs are strongly associated with malignant PC progression. GSVA results revealed that signaling pathways associated with tumor malignant progression (cell cycle, DNA replication, PC, and regulation of actin cytoskeleton) were markedly widened in high-risk individuals. DRGs sensitize the actin cytoskeleton to disulfide bonds stress, leading to actin network collapse and cell death, confirming the potential association between this prognostic signature and disulfidptosis. In addition, patients at low risk showed greater susceptibility to cisplatin, gemcitabine, irinotecan, olaparib, and oxaliplatin, which are synergistic agents or multidrug combination options suggested for PC treatment in combination with gemcitabine^17^. The model construction for this study identified ten signature genes that include MET, ANLN, ANXA3, NT5E, IGF2BP3, FAM83A, RTN1, AREG, DSG3, and KRT6A. Among these genes, MET (mesenchymal-epithelial transition protein) is a receptor tyrosine kinase involved in several aspects of epithelial carcinogenesis, including PC development. Its overexpression impairs patient prognosis, and as such, it is considered a promising therapeutic target for PC^18,19^. In several cellular, animal, and preclinical studies, MET inhibitors have demonstrated preliminary therapeutic effects by impairing epithelial mesenchymal transformation (EMT), metastasis, and chemotherapy resistance in PC. Furthermore, they enhance the effects of radiotherapy and anti-PD-1/PD-L1 treatments^20–25^. ANLN (Anillin), an actin-binding protein, is dysregulated in expression in various tumors, including PC, and is involved in tumor growth and metastasis^26,27^. For instance, Wang et al. found that ANLN-induced EZH2 upregulation mediated the miR-218-5p/LASP1 signaling axis to promote PC progression^26^. Annexin A3 (ANXA3) expression levels were higher in PC patients carrying the H2BG53D mutation and were associated with worse patient outcomes^28^. NT5E (5'-Nucleotidase Ecto, CD73) is a transmembrane glycoprotein that promotes the initiation, metastasis, chemotherapy resistance, angiogenesis, and immune evasion of various cancers^29–34^. NT5E is overexpressed in the cytoplasm of PC cells and promotes EMT and metastasis by competing with Snail for binding to the E3 ligase TRIM21, leading to the inhibition of Snail ubiquitination and degradation^35^. IGF2BP3 (Insulin-like growth factor 2 mRNA binding protein 3) was initially found to be overexpressed in PC^36^. More recent studies have shown that the METTL3-IGF2BP3 axis regulates m6A modification of Spermine synthase (SMS) to promote the capability of PC cells to proliferate and migrate through PI3K-AKT/EMT signaling pathway^37^. FAM83A (Family with sequence similarity 83 member A) is known as oncogenic in several cancer types^38–40^. Recent studies have shown that FAM83A induces EMT through the PI3K/AKT and ERK pathways to promote the development of pancreatic neuroendocrine tumor^41^. RTN1, which is a reticulon protein family member, has been considered a novel tumor suppressor in gastrointestinal mesenchymal tumors^42^ and is associated with a good prognosis in B-cell lymphoma^43^. Similarly, our findings show that RTN1 expression is negatively associated with the risk of PC patients. AREG, a recently discovered molecule, mediates EMT of PC via the EGFR/ERK/NF-κB signaling pathway, which promotes PC growth and metastasis^44^. DSG3 is involved in numerous cell biology functions (cell growth and differentiation)^45^, is considered a negative prognostic biomarker for resected PC^46^ and promotes PC tumorigenicity through the activation of the Src-FAK signaling pathway^47^. This study found that DSG3 was associated with higher clinical staging in PDAC patients and that its high expression predicted a worse outcome in PDAC patients. DSG3 was an independent risk factor for the prognosis of PDAC patients. In addition, DSG3 was found to be significantly associated with the level of multiple immune cell infiltration. This study provides experimental evidence demonstrating that DSG3 is upregulated in PDAC tissues and significantly promotes the migration and invasion of PDAC cells. KRT6A, an essential cytoskeletal component in mammalian cells, mediates alterations in tumor-associated macrophage (TAMs) subtypes through different proteins and pathways in PC and is considered a new potential therapeutic target for TAMs in PC immunotherapy^48^.

KRAS, TP53, CDKN2A, and SMAD4 are commonly known as driver genes in PC (PC), frequently bearing mutations^49^. Despite extensive studies that have confirmed the influence of driver genes on most aspects of PC development, novel regulatory mechanisms continue to emerge as high-throughput and multi-omics technologies advance. Mutations in KRAS and TP53 are closely related to cell death^50^. TP53 can act as a transcription factor to promote apoptosis by transactivating pro-apoptotic genes (BAX, BBC3, and PMAIP1/NOXA)^51–53^. Alternatively, TP53 regulates non-apoptotic regulated cell death (RCD) types, like ferroptosis. TP53 promotes ferroptosis by transcriptionally repressing SLC7A11/xCT expression in lung cancer cells, while the TP53P47S variant suppresses SLC7A11 to a lesser extent, leading to tumor development^54–56^. The crucial role of SLC7A11 in disulfidptosis and whether TP53 mutations can regulate SLC7A11 to participate in disulfidptosis regulation is uncertain. Over 95% of PDAC patients have mutated KRAS, and KRAS-mediated downstream pathways are essential in initiating and maintaining PDAC^57,58^. RAS mutations, including KRAS, sensitize cancer cells, including pancreatic ductal adenocarcinoma (PDAC) cells, to ferroptosis induction due to mutant RAS-mediated expression of iron metabolism genes, such as Tfrc, Fth1, and Ftl^59^. Overexpression of SLC7A11 in KRAS-mutant lung adenocarcinoma (LUAD) patients is linked to increased susceptibility to SLC7A11 inhibition in cells carrying KRAS-mutant LUAD, which significantly reduces cystine uptake and intracellular glutathione biosynthesis^60^. Cysteine is a vital element in the development of disulfidptosis. However, whether PDAC cells with KRAS or other RAS mutations show different sensitivities to disulfidptosis warrants further investigation.

TME is a current area of intense research due to its strong association with cancer development, therapy resistance, and prognosis^61,62^. Several studies have reported a positive prognostic outcome in patients with malignancies who exhibit B-cell infiltration^63–65^. In this study, a similar conclusion was reached regarding the higher levels of B-cell infiltration observed in low-risk PC patients with better prognoses. While CD8 + T cells can selectively target and obliterate cancer cells, they are often in a dysfunctional state in tumors^66^. Various factors can cause CD8 + T cell dysfunction, such as tumor cells, M2 macrophages, MDSCs, among others^66^. The higher level of M2 macrophage infiltration observed in this study may have contributed to a low infiltration level of CD8 + T cell in high-risk individuals. Higher levels of infiltration of T cells CD8, T cells CD4 memory resting, monocytes, plasma cells, and dendritic cells resting were shown in cluster B and low-risk patients, indicating a supportive prognostic role for these cells in PC. TAMs are related to inferior outcome and rapid disease progression in many types of cancer, including PC, and are thought to impair tumor-specific immune responses, leading to a more aggressive malignant phenotype^67,68^. In PC, the infiltration of TAMs has been shown to inhibit gemcitabine through the release of pyrimidines, leading to gemcitabine resistance in patients^69^. Another study found that TAM-derived exosomes can induce tumor resistance to cisplatin^70^. In addition to TAMs, this study also revealed that high-risk PC patients have significantly elevated levels of immune checkpoint expression, including PD-L1. Immune checkpoints expressed by tumor cells can have an immunosuppressive effect on immune cells, and PD-L1 has been shown to play a pivotal role in tumor cell immune escape. Although blocking the binding between PD-1 with PD-L1 can enhance immune function and demonstrate sustained tumor regression and disease stabilization in various advanced cancers^71^, single-agent PD-L1 inhibitor treatment has not been effective in PC^72–74^. Therefore, the search for a breakthrough in anti-PD-L1-based combination therapy has become a primary research focus to overcome this dilemma. Combination regimens, including chemotherapy (such as gemcitabine), radiotherapy, other locoregional therapies, molecularly targeted therapy, and immunotherapy, have emerged and have shown partially promising results in cell, mouse models, and preclinical studies. However, their clinical application is far from satisfactory^74^. Therefore, the development of novel and effective agents or combination modalities is imminent.

Despite the significant findings in this study, several limitations exist. First, the DRGs were derived from a limited literature, and it is possible that other potential DRGs have not been included. Second, the mechanisms underlying the potential association between the 10 signature genes with disulfidptosis in PC require confirmation through in-depth studies. Third, the stability and reliability of the prognostic signature requires further validation in large prospective studies at PC research centers. Future studies should aim to address these limitations to improve the overall quality and applicability of the findings.

## Conclusion

The present study has developed a new prognostic signature and molecular subtype for PC (PC) patients based on differentially regulated genes (DRGs). This approach provides a novel perspective for categorizing and treating patients with PC, and can aid in predicting patient prognosis. Furthermore, the study highlights the potential value of variations in the immune cells in TME for prognosis and therapy of PC patients. Future research can further validate and expand on these findings to enhance the understanding and management of PC.

### Supplementary Information

Supplementary Information.

Supplementary Table S1.

## Data Availability

The data used to support the findings of this study are available from the corresponding author upon request.
