# Supplementary figures and images for: Integrated single-cell and bulk RNA sequencing in pancreatic cancer identifies disulfidptosis-associated molecular subtypes and prognostic signature
Source: Sci Rep. 2023 Oct 16;13:17577. doi: 10.1038/s41598-023-43036-7 (PMC10579418; doi:10.1038/s41598-023-43036-7)

## 01.PDAC tissue-WB

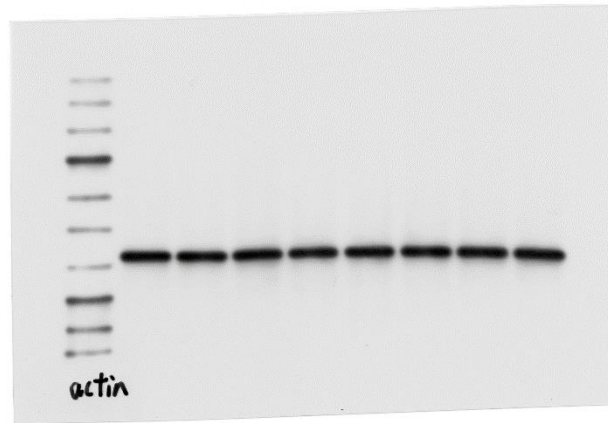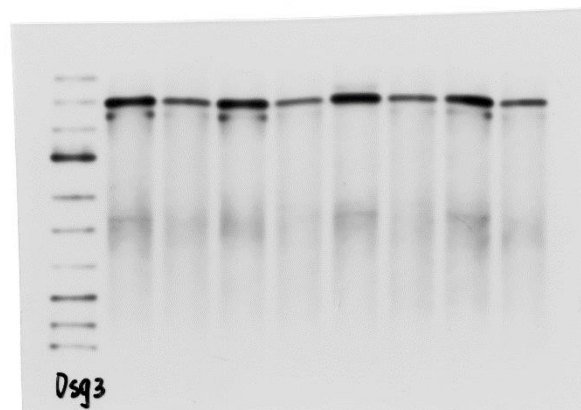

## 02.knockdown-WB

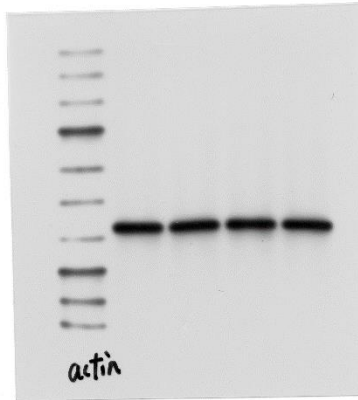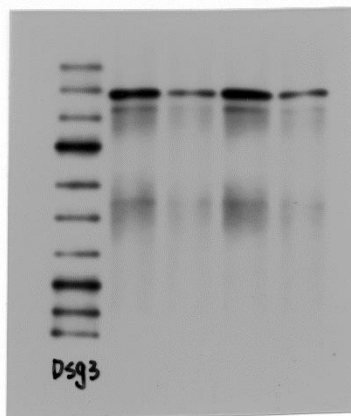

03.overexpression-WB

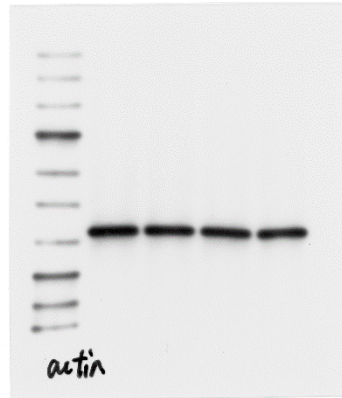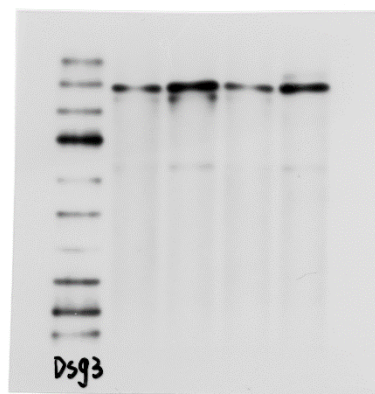

Supplement: Supplementary file 1 — Supplementary Information. [file 41598_2023_43036_MOESM1_ESM.pdf]
