# Supplementary material for: Integrated single-cell and bulk RNA sequencing in pancreatic cancer identifies disulfidptosis-associated molecular subtypes and prognostic signature
Source: Sci Rep. 2023 Oct 16;13:17577. doi: 10.1038/s41598-023-43036-7 (PMC10579418; doi:10.1038/s41598-023-43036-7)
Supplement: Supplementary file 2 — Supplementary Table S1. [file 41598_2023_43036_MOESM2_ESM.docx]

**Table S1** The list of 223 DEGs between cluster B with clusters A/C/D.

| DEGs |
| --- |
| AHNAK2 |
| SLC7A11 |
| MYOF |
| ASAP2 |
| MALL |
| ITGA2 |
| INPP4B |
| MET |
| LMO7 |
| ADAM9 |
| ANXA2 |
| PLEC |
| FLNB |
| FAT1 |
| SLC2A1 |
| PLEK2 |
| TNFRSF21 |
| ANLN |
| MMP14 |
| HK2 |
| COL17A1 |
| SERPINB5 |
| TMC7 |
| GPRC5A |
| ANO1 |
| ITGB4 |
| S100A16 |
| ITPR3 |
| ANXA3 |
| EPHA2 |
| ABHD17C |
| PLS1 |
| GALNT5 |
| FA2H |
| LAMB3 |
| SDR16C5 |
| LAMC2 |
| NT5E |
| CD55 |
| STYK1 |
| FERMT1 |
| SCEL |
| TGFA |
| S100A6 |
| IGF2BP3 |
| B3GNT3 |
| MST1R |
| S100A11 |
| LGALS3 |
| GJB3 |
| KLF5 |
| LAMA3 |
| PTPRR |
| TSPAN1 |
| FUT3 |
| KRT19 |
| FBLL1 |
| CDH3 |
| MAL2 |
| ITGB6 |
| GJB2 |
| PPARG |
| PTK6 |
| TMPRSS4 |
| TSPAN7 |
| S100P |
| HKDC1 |
| CD109 |
| RUNDC3A |
| FAM83A |
| TM4SF1 |
| GJB4 |
| FOXL1 |
| SCNN1A |
| MSLN |
| S100A14 |
| KCNN4 |
| CEMIP |
| SERINC2 |
| PCDH7 |
| F3 |
| RTN1 |
| XDH |
| SFN |
| VILL |
| UGT1A10 |
| PLAU |
| LIPH |
| IL1RN |
| MUC5AC |
| MYEOV |
| EREG |
| DKK1 |
| SEMA7A |
| CEACAM6 |
| LINC01559 |
| MXRA5 |
| ADAMTS12 |
| BCAS1 |
| ACSL5 |
| ECM1 |
| PITX1 |
| TNS4 |
| APLP1 |
| AREG |
| EPS8L1 |
| B3GNT7 |
| SOX21 |
| PRSS8 |
| PHLDA2 |
| POF1B |
| FER1L6 |
| FN1 |
| TGFBI |
| MROH6 |
| PADI1 |
| BEX2 |
| ANXA10 |
| AGR2 |
| KRT7 |
| LEMD1 |
| EVPL |
| THBS2 |
| TRIM29 |
| KLK7 |
| KCNK3 |
| CEACAM5 |
| TMC5 |
| ST6GALNAC1 |
| WNT7A |
| CA12 |
| ARL14 |
| B3GNT6 |
| SPDEF |
| MIR7-3HG |
| CYP2S1 |
| CTSE |
| COL5A1 |
| KRT16 |
| SLC6A20 |
| KLK10 |
| SFTA2 |
| COL1A1 |
| TSPAN8 |
| COL12A1 |
| COL11A1 |
| MIR3682 |
| EGLN3 |
| PCSK1N |
| NKX2-2 |
| CLIC3 |
| SCG3 |
| DSG3 |
| COL7A1 |
| DHRS9 |
| PDZK1IP1 |
| TNNT1 |
| CDHR2 |
| PTGES |
| MUC1 |
| COL1A2 |
| NMU |
| CALB2 |
| MMP11 |
| CDA |
| TACSTD2 |
| SCG5 |
| MUC4 |
| PLAT |
| COL5A2 |
| VSIG2 |
| SULF1 |
| COL3A1 |
| KLK6 |
| CAPN9 |
| PROM2 |
| PSCA |
| COL10A1 |
| ERN2 |
| NTSR1 |
| ABCC8 |
| CA9 |
| BEX1 |
| MUC16 |
| MUC17 |
| CAPN8 |
| APOH |
| LGALS4 |
| EPS8L3 |
| SCGN |
| KRT6A |
| LCN2 |
| TFF1 |
| KRT17 |
| PCSK2 |
| IGHD |
| POSTN |
| CST6 |
| FGFBP1 |
| VSIG1 |
| AGR3 |
| MMP1 |
| MUC5B |
| CRABP2 |
| S100A2 |
| MMP7 |
| SPRR1B |
| CHGB |
| SPRR3 |
| TCN1 |
| CLDN18 |
| FDCSP |
| TFF2 |
| MT-TY |
| CXCL5 |
| LY6D |
| SFRP2 |
| REG4 |
| CCL19 |
| CHGA |
| SPRR1A |
| TNFRSF6B |
| PPY |
